# Treatable causes of fever among children under five years in a seasonal malaria transmission area in Burkina Faso

**DOI:** 10.1186/s40249-018-0442-3

**Published:** 2018-05-31

**Authors:** Francois Kiemde, Marc Christian Tahita, Palpouguini Lompo, Toussaint Rouamba, Athanase M. Some, Halidou Tinto, Petra F. Mens, Henk D. F. H. Schallig, Michael Boele van Hensbroek

**Affiliations:** 1 Institut de Recherche en Science de la Sante-Unite de Recherche Clinique de Nanoro, Nanoro, Burkina Faso; 2 Academic Medical Centre, Department of Medical Microbiology, Parasitology Unit, Amsterdam, The Netherlands; 3 Global Child Health Group, Academic Medical Centre, University of Amsterdam, Amsterdam, The Netherlands

**Keywords:** Fever, Children, Infectious diseases, Malaria

## Abstract

**Background:**

Fever remains a major public health problem. In Burkina Faso, more than half of febrile children are considered not to be infected by malaria. This study prospectively assessed probable (treatable) causes of fever in Burkinabe children.

**Methods:**

A prospective study was conducted among febrile children (≥37.5 °C) under 5 years of age presenting at four health facilities and one referral hospital in rural Burkina Faso. From each participant, blood was collected for malaria microscopy and culture, urine for dipstick testing and culturing if tested positive for leucocytes and nitrite, stool for rotavirus/adenovirus testing, culture and parasitology, and a nasopharyngeal swab for culture.

**Results:**

In total 684 febrile children were included in the study. *Plasmodium falciparum* malaria was found in 49.7% (340/684) of the participants and non-malaria infections in 49.1% (336/684) of children. The non-nalaria infections included gastro-intestinal infections (37.0%), common bacterial pathogens of nasopharynx (24.3%), bacterial bloodstream infections (6.0%) and urinary tract infections (1.8%). Nearly 45% (154/340) of the malaria infected children were co-infected with non-nalaria infections, but only 3.2% (11/340) of these co-infections could be considered as a possible alternative cause of fever. In contrast, in the malaria microscopy negative children 18.0% (62/344) of the infections could be the probable cause of the fever. Pathogens were not isolated from 23.7% (162/684) of the febrile cases.

**Conclusions:**

Malaria remains the most common pathogen found in febrile children in Burkina Faso. However, a relative high number of febrile children had non-malaria infections. The correct diagnosis of these non-malaria fevers is a major concern, and there is an urgent need to develop more point-of-care diagnostic tests and capacities to identify and treat the causes of these fevers.

**Electronic supplementary material:**

The online version of this article (10.1186/s40249-018-0442-3) contains supplementary material, which is available to authorized users.

## Multilingual abstract

Please Additional file 1 for translation in the five official working languages of the United Nations.

## Background

Febrile illnesses remain a major public health problem in Sub-Saharan Africa (SSA). Fever is the most common clinical symptom found in children under five years of age presenting at health facilities [1, 2]. Nowadays in many malaria endemic areas, including Burkina Faso, more than half of these febrile children are considered not to be infected with malaria, as the incidence of this disease is declining due to increased control efforts over the last decade [3–6]. This, combined with the guidelines of the World Health Organization (WHO) that recommend to confirm a malaria infection in febrile children through a diagnostic test before giving antimalarial treatment [7], prompts the need to search for an alternative cause of fever. This has created a diagnostic dilemma for health workers, by having a large group of patients with so called “unexplained” or “non-malaria” fever and with few or no diagnostic tools available to guide the subsequence management of these febrile cases [8–10]. An alternative cause of fever may also be present in malaria positive children. Subsequently, lack of knowledge on the prevalence of other possible aetiologies of fever, limited access to proper diagnostic tests, as well as the fear of overlooking a potentially life threatening malaria infection are the main reasons why most febrile children still receive antimalarial treatment [11–13].

The various possible aetiologies of a febrile illness are difficult to distinguish when only information from medical history and physical examination are available [14, 15]. Laboratory facilities are often scarce in health facilities in low-and middle-income countries [11, 16, 17]. Therefore, fever is routinely treated in these countries using only clinical signs and symptoms (empiric treatment) [18, 19]. Data are becoming available on the aetiologies of fever episodes in children under 5 years of age in SSA [20–24]. It is noted that, next to malaria, viral infections are a very common cause of febrile disease [13, 21, 23], which cannot be treated with antibiotics. However, a significant number of febrile cases remain that do need treatment with appropriate antibiotics, but these treatable infections are often overlooked [20, 22]. The clinician needs to know the possible presence of other treatable infections amongst malaria-infected and uninfected children presenting with fever.

The present prospective study was designed and conducted to identify the pathogens that could cause fever in children seeking care at five health centres in rural Burkina Faso. Febrile patients were evaluated for a wide range of infections, with a focus on treatable bacterial and parasitic infections, using conventional diagnostic. This study contributes to a better understanding of possible causes of fever in children under 5 years of age living in a malaria endemic region.

## Methods

### Study site

This study was conducted in the rural district of Nanoro, Burkina Faso. Four peripheral health facilities (Urbain, Godo, Nazoanga and Seguedin) and the referral hospital Centre Medical Saint Camille of Nanoro were involved in the data and clinical specimen collection. The choice of the peripheral health facilities was based on their accessibility for patients and their distance to the research laboratory (maximum of 20 km). The district hospital was included as recruitment centre in order to be able to capture also the more severely ill children who were referred from peripheral health facilities.

Malaria transmission in Nanoro district is hyper endemic and occurs principally between July and November. In 2010, the average under five mortality rate in Burkina Faso was 129/1000 life-births [25]. Vaccination against *Haemophilus influenzae* type b was introduced into the extended program of immunization (EPI) in January 2006 (estimated coverage 86.8% in 2012 [26]) and against pneumococcal and rotavirus in October 2013 (Source: Ministry of Health, Burkina Faso).

### Study design

A prospective study was conducted from January–December 2015. The study was approved by the National Ethical Committee in Health Research, Burkina Faso (Deliberation N°2014–11-130). All children under 5 years of age with an axillary temperature ≥ 37.5 °C presenting at one of the participating health facilities or the referral hospital were asked to participate in the study. Written informed consent was obtained from parent or legal guardian prior to enrolment. The medical history and findings from clinical examination of the child were recorded on a standard Case Report Form (CRF) by trained study nurses. The following clinical specimens were collected from each participant: blood, urine, stool and a nasopharyngeal swab. Parents or adult guardians were asked to complete sample collection within 48 h following inclusion when diagnostic specimen collection (stool and/or urine) could not be realized during the enrolment procedure. They were provided with instructions and sterile containers for that purpose. The parents or guardians were told to return the samples immediately after collection.

The participants were managed according to the national guidelines. If culture results or other laboratory tests justified additional or alternative treatment, the results were forwarded to the health facility. The parents or guardians were contacted as soon as possible to arrange that the necessary treatment would be given to the participant.

### Laboratory procedures

Nasopharyngeal swab and blood samples were also collected in Skim Milk-Tryptone-Glucose-Glycerol (STGG) and Ethylene Diamine Tetra-Acetic Acid (EDTA) tube respectively. All samples were transported to the central microbiology laboratory of the Clinical Research Unit of Nanoro (CRUN) where the various laboratory tests were conducted.

#### Malaria testing

Giemsa stained thick and thin blood smears were prepared and independently read by two experienced microscopists. In case of discordance (i.e. positive vs. negative, difference in *Plasmodium* species, difference in parasite density > Log10 or ratio > 2 in case of parasite density ≤ 400/μl or > 400/μl) the respective slide was read by a third independent reader to make a final decision. The final parasite counts were the geometric means of the two reader’s results or the geometric means of the two geometrically closest reading in case of third reading. These results were expressed as asexual parasites per microliter by using the patient’s white blood cell (WBC) count.

#### Full blood counts

Blood samples were collected in EDTA tubes for full blood counts using a Sysmex XS1000i (Sysmex Corporation, Kobe, Japan) according to manufacturer’s instructions.

#### Blood culture

From each child, 1–3 ml of venous blood was directly collected in a paediatric blood culture bottle (BD BACTEC Peds Plus™/F, Becton Dickinson and Company, Sparks, Maryland, USA) and incubated in a BACTEC 9050 instrument (Becton Dickinson) for a total of 5 days. If flagged for bacterial growth, the cultures were subsequently Gram stained, sub-cultured on Eosin-Methylene Blue (EMB) agar, 5% Sheep Blood agar (bioMérieux, Marcy-l’Etoile, France) and chocolate + isovitalex agar, and incubated at 35–37 °C for 24 h. Isolates were identified by standard microbiological methods.

#### Stool examination

Fresh stool smears were prepared in normal saline and examined by direct microscopy for the presence of intestinal parasites. Parasite elements looked for were: cysts, eggs, and vegetative forms of protozoa. Furthermore, stools were plated on EMB, Hektoen agar and inoculated in selenite of sodium broth and incubated at 35–37 °C. After 4 h of incubation, selenite of sodium broth was sub-cultured on *Salmonella* and *Shigella* agar (*SS* agar). To assess the prevalence of rotavirus and adenovirus after the introduction of vaccine against rotavirus in 2013, stool samples were also analysed for group A rotavirus using one step rotavirus and adenovirus serotype 40/41 in human faeces (SD Bioline Rota/Adeno; Standard Diagnostics Inc., Korea).

#### Urine examination

Dipstick testing (UroColor, Standard Diagnostics Inc., Korea) was done for each urine sample and the parameters collected were: presence or not of blood, protein, leucocytes and nitrite in the sample. A culture of the sample was done in case testing for leucocytes and nitrate was positive. Urine was plated on Cystine Lactose Electrolyte Deficient and EMB agar when dipstick testing for leucocytes and nitrite was positive. The cultures were incubated at 35–37 °C for 24 h. Only samples that yielded pure bacterial growth of more than 10^5^ colonies forming units (CFU)/ml were considered significant bacteriuria.

#### Nasopharyngeal specimen culture

A flexible flocked swab was introduced into the nasal passage and two rotations of swabbing were done. The swab was placed into STGG broth for storage and transportation. For analysis, nasopharyngeal swab was vortexed and 200 μl of this broth was inoculated in 10 ml of Todd-Hewitt (TH) broth for enrichment and incubated at 35–37 °C during 18 to 24 h. The TH broth was subsequently plated out and incubated at 35–37 °C for 24 h on sheep blood agar and chocolate + isovitalex agar and chapman (mannitol-salt) agar to detect *Streptococcus* and/or *Staphylococcus* species, respectively.

#### Interpretation of laboratory findings

For the purpose of this study, the infections found, except malaria, were considered to be non-malaria infections. The non-malaria infections were further classified as either “probable causes of fever” or “non-probable causes of fever”. The infections that could be considered as a “probable cause of fever” in the recruited cases were subsequently further regrouped in: bacterial bloodstream infections (bBSI), viral gastro-intestinal (vGII) caused by rotavirus or adenovirus, and urinary tract infections (UTI) [27–30]. The infections that could be considered as “non-probable cause of fever” in the study cases, were regrouped in: bacterial gastro-intestinal infection (bGII), common bacterial pathogens of the nasopharynx (CBPN) and parasitic gastro-intestinal infection (pGII), except for amoeba.

## Data analysis

Data management involved double data entry using Open Clinica software. Data analysis was done by using R software version 3.3.1 (R development core team 2016, R Foundation for Statistical Computing, Vienna, Austria). Categorical variables were summarized as proportions and Pearson’s Chi-square test or Fisher’s exact test were performed. Continuous variables were described by mean or median and compared by using the Student’s *t*-test. Binomial logistic regression was used to compare the relation between the presence of infection and demographic, clinical and laboratory data. The *P* value < 0.05 was considered as significant.

## Results

### Characteristic of the study population

**Fig. 1:**
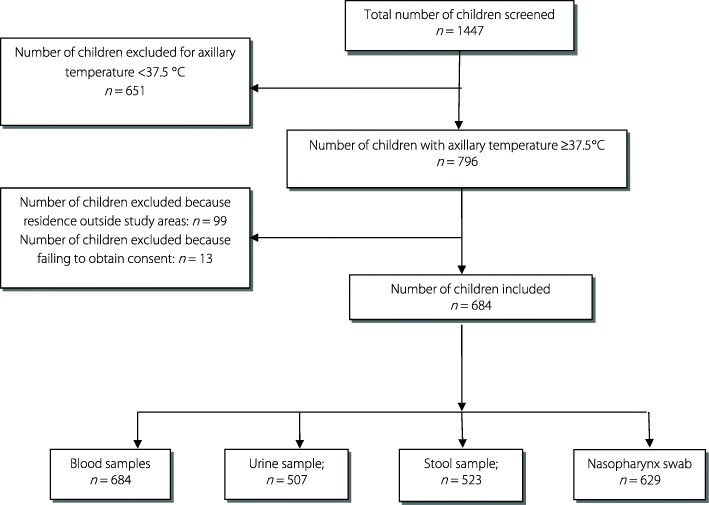
Study flow chart

A total of 1447 children under 5 years of age, attending one of the participating health facilities or the referral hospital during the year 2015, were screened. 796 (55.0%) children were febrile (axillary temperature ≥37.5 °C) and 684 febrile children (85.9%) were included in the study. The study flow chart is presented in Fig. 1. Reasons for exclusion were: residence outside the health facilities catchment areas or failing to obtain consent from parents or guardians. The mean age of the recruited children was 22.4 months (Standard deviation [*SD*] = 14.1) and 29.4% were under 12 months of age. Mean axillary body temperature was 38.7 °C (37.5–41.0 °C) (Table 1).

**Table 1:** Demographic characteristics, clinical symptoms and basic laboratory findings of study subjects enrolled in the present study in four rural health centres and one referral hospital in Nanoro Health district

Category	Subcategory	*N*	Proportion (%)
Gender	Male	369	53.95
	Female	315	46.05
Age	≤12 months	201	29.39
	>12 months	483	70.61
Temperature	≥37.5 °C–≤38.5 °C	340	49.71
	>38.5 °C–≤39.5 °C	242	35.38
	>39.5 °C	102	14.91
Duration of fever	1 day	108	15.79
	2–3 days	518	75.73
	4–7 days	56	8.19
	>7 days	2	0.29
Vaccination according to the EPI	Yes	449	65.64
	No	133	19.44
	Unknown	102	14.91
Common symptoms	Cough	335	48.98
	Diarrhoea	266	38.89
	Vomiting	123	17.98
	Others	55	8.04
Basic laboratory findings	Haemoglobin rate		
	< 8 g/dl	180	26.32
	≥ 8 g/dl–<11 g/dl	418	61.11
	≥11 g/dl	86	12.57
	White blood cells		
	<4 × 10^3^ cells/mm^3^	10	1.46
	≥4 ×10^3^ cells/mm^3^–< 12 × 10^3^ cells/mm^3^	348	50.88
	≥12 × 10^3^ cells/mm^3^	326	47.66

*EPI* Expanded program of immunization

Clinical specimens for blood culture and malaria microscopy were obtained from all study cases. The proportion of stool and urine samples collected was 76.5% (523/684) and 74.1% (507/684), respectively. A small proportion (61/684, 8.9%) of nasopharynx cultures could not be performed, because the training of study nurses to collect nasopharyngeal swabs was completed after the start of inclusion (Fig. 1 and Table 2).

**Table 2:** Microbiology and malaria microscopy findings of children under-5 years enrolled in the study

Subcategory	General population *n*/*N* (%)	Referral hospital *n*/*N* (%)	Health facilities *n*/*N* (%)
Malaria microscopy	684/684 (100)	138/138 (100)	546/546 (100)
Malaria microscopy positive	340/684 (49.71)	41/138 (29.71)	299/546 (54.76)
*Plasmodium falciparum*^a^	340/340 (100)	41/138 (29.71)	299/546 (54.76)
*Plasmodium malariae*	04/340 (0.88)	0/138 (0)	4/299 (1.34)
*Plasmodium ovalae*	01/340 (0.29)	0/138 (0)	1/299 (0.33)
Blood cultures (*N* = 684)	684/684 (100)	138/138 (100)	546/546 (100)
Bacterial culture positive	41/684 (5.99)	21/138 (15.22)	20/546 (3.66)
*Non-Typhoid Salmonella* spp.	31/41 (75.61)	19/21 (90.48)	12/20 (60.00)
*Salmonella typhi*	02/41 (4.88)	00	02/20 (10.00)
*Escherichia coli*	01/41 (2.44)	00	01/20 (5.00)
*Staphylococcus aureus*	02/41 (4.88)	01/21 (4.76)	01/20 (5.00)
*Streptococcus pneumoniae*	03/41 (7.31)	01/21 (4.76)	02/20 (10.00)
*Neisseria meningitidis*	02/41 (4.88)	00	02/20 (10.00)
Urine examination (*N* = 507)	507/684 (74.12)	94/138 (68.12)	413/546 (75.64)
Bacterial culture positive	10/507 (1.97)	04/94 (4.26)	06/413 (1.45)
*Escherichia coli*	10/10 (100.00)	04/4 (100)	06/6 (100)
Stool examinations (*N* = 523)	523/684 (76.46)	85/138 (61.60)	438/546 (80.22)
Bacterial culture positive	40/523 (7.65)	09/85 (10.59)	31/438 (7.08)
*Non-Typhoid Salmonella*	20/40 (50.00)	08/9 (88.89)	16/31 (38.71)
*Escherichia coli*	17/40 (42.50)	01/9 (11.11)	16/31 ()51.61
*Shigella*	03/40 (7.50)	00	03/31 (9.69)
Parasites positive	143/523 (27.34)	17/85 (20.00)	126/438 (28.77)
Mono parasitosis^b^	115/143 (80.42)	11/17 (64.71)	104/126 (82.54)
Bi parasitosis	25/143 (17.48)	05/17 (29.41)	20/126 (15.87)
Tri parasitosis	03/143 (2.10)	01/17 (5.88)	02/126 (1.59)
Rotavirus/Adenovirus positive	25/523 (4.78)	04/85 (4.71)	21/438 (4.80)
Rotavirus	15/25 (60.00)	00	15/21 (71.43)
Adenovirus	10/25 (40.00)	04/4 (100)	06/21 (28.57)
Nasopharyngeal cultures (*N* = 629)	629/684 (91.95)	137/138 (99.28)	492/546 (90.11)
Bacterial culture positive	153/629 (24.32)	41/137 (29.71)	112/492 (22.77)
*Staphylococcus aureus*	148/153 (96.73)	41/41 (100)	107/112 (95.54)
*Streptococcus pneumoniae*	05/153 (3.27)	00	05/112 (4.46)
No pathogens detected	162/684 (23.68)	40/138 (28.99)	122/546 (22.34)

^a^: 5 children infection with *Plasmodium falciparum* were co-infected with *P. malariae* and *P. ovale*

^b^: *Giardia intestinalis*: 35.65%; *Trichomonas intestinalis*: 22.61%; *Endolimax nana*: 17.40%; *Entamoeba coli*: 13.91%; *Entamoeba hystolitica*: 7.82%; Others: 2.61%

### Prevalence of malaria and other pathogens in the febrile study cases

**Fig. 2:**
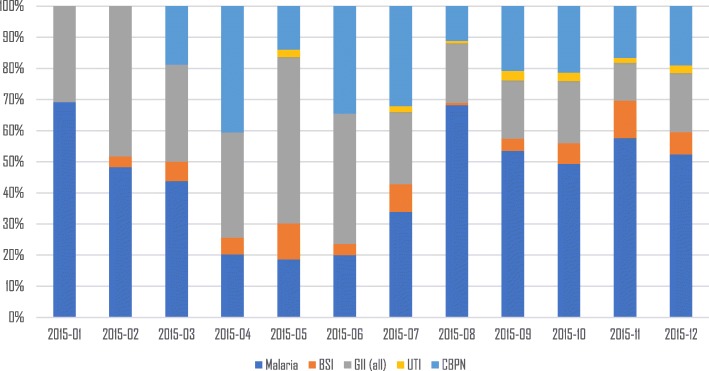
Distribution of pathogens found with microbiology during the whole study (year 2015). *BSI* Bloodstream infection (bacterial), *GII* Gastro-intestinal infection (all), *UTI* Urinary tract infection, *CBPN* common bacterial pathogens of nasopharynx

Malaria (confirmed by positive microscopy) was found in 49.7% (340/684) of the cases. Malaria occurred whole year round (Fig. 2). All 340 malaria microscopy positive cases were infected with *Plasmodium falciparum,* but five children were co-infected with other *Plasmodium-*species (i.e. 4 with *P. malariae* and 1 with *P. ovale*). The geometric mean parasite density was 24 003 parasites/μl (range: 72–2 272 500). Malaria was more prevalent in children presenting at the health facilities than at the referral hospital (Table 2).

Bacteria could be identified in 6.0% (41/684) of blood cultures, 2.0% (10/507) urine cultures, 7.7% (40/523) stools and 24.3% (154/623) nasopharyngeal cultures. Parasites were found in 27.3% (143/523) of the collected stool specimen and 4.8% (25/523) of these samples were positive for rotavirus/adenovirus. Pathogens could not be detected in 162 children (23.7% of the study population) (Table 2).

A pathogen (other than malaria) was identified in 10.7% (73/684) of the febrile children that could be “a probable cause of fever” (Table 2). Of these pathogens, 6.0% (41/684) was a bacterial bloodstream infection (bBSI), 4.8% (25/253) was a viral gastro-intestinal tract infection (vGII) and 2% (10/507) was a urinary tract infection (UTI). Only mono-infections were found in the bacterial cultures (bBSI and UTI). Non-typhoid *Salmonella* was the dominant bacterium isolated from positive blood cultures (75.6%, 31/41) and all ten positive urine cultures contained *Escherichia coli* (10/10). Rotavirus was the main virus detected in positive stools tested with rotavirus/adenovirus tests (60%, 15/25). Pathogens that could be “a probable cause of fever” (bBSI and UTI, except for vGII) were more often found in clinical specimen from children presenting at the referral hospital than in those presenting at the peripheral health facilities (Table 2).

In 40.8% (279/684, i.e. 143 children in the malaria microscopy positive group and 136 children in the malaria microscopy negative group) of the febrile children one or more pathogens were identified that could be considered as “non-probable cause of fever” (Table 3). Parasites were detected in 27.3% (143/523) of the stool samples. *Giardia intestinalis* was the most frequently found parasite in stool samples 46.2% (66/143). Pathogenic bacteria were isolated from 7.7% (40/523) of the stool samples (Table 2). The most often isolated bacterial species from stool was non-typhoid Salmonella 50.0% (20/40). Nasopharyngeal swab cultures were positive for 24.3% (153/629) of the febrile cases. *Staphylococcus aureus* was the most frequently found species 96.7% (148/153) amongst the common bacterial pathogens of nasopharynx (CBPN). The prevalence of pathogens that were probably not the cause of fever was more or less similar in clinical specimen from children presenting at the referral hospital and in those presenting at the peripheral health facilities (see Table 2).

**Table 3:** Interaction between malaria and others pathogens studied

	Malaria microscopy positive (340) *n* (%)	Malaria microscopy negative (344) *n* (%)
Other infections	154 (45.29)	182 (52.91)
Probable cause of fever (BSI, vGII, UTI)	11 (3.23)	62 (18.02)
No probable causes of fever (pGII, bGII, CBPN)	143 (42.05)	136 (39.53)
bBSI	6 (1.76)	35 (10.17)
GII (all)	90 (26.47)	103 (29.94)
pGII	77 (22.64)	66 (19.18)
bGII	17 (5.0)	23 (6.69)
vGII	3 (0.88)	22 (6.40)
CBPN	75 (22.05)	78 (22.67)
UTI	3 (0.88)	7 (2.03)

*bBSI* bacterial bloodstream infection, *GII* Gastro-intestinal infection, *pGII* parasitic gastro-intestinal infection, *bGII* bacterial gastro-intestinal infection, *vGII* viral gastro-intestinal infection, *UTI* Urinary tract infection, *CBPN* Common bacterial pathogens of nasopharynx

All pathogens were found the whole year round except for UTI. Malaria followed more or less the typical seasonal distribution for the study area, with peak transmission between August and January (Fig. 2). CBPN were not studied during the first 2 months of the study. UTIs were found mainly during the second half of the year (Fig. 2).

### Interaction between malaria and other pathogens

Additional pathogens, next to *Plasmodium,* were isolated from 45.3% (154/340) of the clinical samples obtained from children who were malaria microscopy positive (Table 3). Only 11/154 (7.1%) of these other pathogens were identified as a pathogen that could also be considered as a “probable cause of fever”. Consequently, 3.2% (11/340) of the malaria microscopy positive children were co-infected with other pathogens that could be a “probable cause of fever”. In the malaria microscopy negative group 52.9% (182/344) of other infections were found. A significant higher number of these pathogens, 18.0% (62/344), was identified as a “probable cause of fever” in the malaria negative group of children compared to the malaria infected cases (*P* < 0.001).

### Correlation between the presence of infection and demographic, clinical and laboratory data

The correlation between the presence of an infection investigated in this study and the patient’s demographic, clinical and laboratory data is summarized in Table 4. The analysis showed a strong correlation between having a malaria infection and age > 12 months (*OR* = 2.85, 95% *CI*: 2.0–4.1, *P* < 0.001) or temperature > 39.5 °C (*OR* = 2.06, 95% *CI*: 1.3–3.3, *P* = 0.002). Furthermore, malaria was associated with white blood cell count > 12 × 10^3^ cells/mm^3^, and haemoglobin concentration < 11 g/dl (see Table 4).

**Table 4:** Correlation between malaria, BSI, UTI, GII and CBPN and demographic, clinical and laboratory data

	Malaria		Blood stream infection		Urinary tract infection		Gastro-intestinal infection		CBPN	
	*OR* (95% *CI*)	*P*-value	*OR* (95% *CI*)	*P*-value	*OR* (95% *CI*)	*P*-value	*OR* (95% *CI*)	*P*-value	*OR* (95% *CI*)	*P*-value
Gender										
Male	1		1		1		1		1	
Female	1.19 (0.88–1.61)	0.255	1.01 (0.53–1.91)	0.969	1.22 (0.34–4.45)	0.752	1.18 (0.83–1.69)	0.350	1.18 (0.82–1.70)	0.381
Age										
≤12 months	1		1		1		1		1	
>12 months	2.85 (2.02–4.06)	<0.001*	0.70 (0.37–1.39)	0.299	0.34 (0.09–1.25)	0.094	2.88 (1.86–4.58)	<0.001*	0.90 (0.61–1.35)	0.609
Temperature										
37.5 °C–38.5 °C	1		1		1		1		1	
38.5 °C–39.5 °C	1.26 (0.91–1.76)	0.166	1.28 (0.64–2.51)	0.479	1.93 (0.50–7.92)	0.330	0.99 (0.67–1.47)	0.963	1.21 (0.81–1.80)	0.334
>39.5 °C	2.06 (1.31–3.26)	0.002*	0.87 (0.28–2.23)	0.789	0.86 (0.04–5.90)	0.891	1.13 (0.67–1.89)	0.647	0.90 (0.50–1.55)	0.714
White cell counts										
4 × 10^3^–12 × 10^3^	1		1		1		1		1	
<4 × 10^3^ cells/mm^3^	1.18 (0.33–4.67)	0.803	0.35 (0.02–7.31)	0.828	0.66 (0.20–26.68)	0.828	1.55 (0.36–6.70)	0.540	1.13 (0.01–2.31)	0.166
≥12 × 10^3^ mm^3^	0.58 (0.43–0.79)	<0.001*	1.03 (0.55–1.91)	0.206	2.25 (0.64–7.92)	0.206	0.79 (0.55–1.14)	0.211	1.36 (0.94–1.95)	0.100
Anaemia										
≥11 g/dl	1		1		1	1	1		1	
<8 g/dl	4.06 (2.35–7.20)	<0.001*	1.60 (0.54–5.80)	0.426	1.50 (0.31–10.70)	0.631	1.71 (0.39–1.28)	0.252	1.16 (0.64–1.18)	0.625
8 g/dl–11 g/dl	2.51 (1.53–4.24)	<0.001*	1.25 (0.47–4.33)	0.688	0.34 (0.06–2.65)	0.248	0.60 (0.35–1.01)	0.052	0.81 (0.46–1.44)	0.453
Vaccination according EPI										
Yes	1		1		1		1		1	
No	0.78 (0.52–1.14)	0.204	0.68 (0.25–1.58)	0.409	0.12 (0.01–2.05)	0.145	1.23 (0.81–1.98)	0.294	0.98 (0.60–1.58)	0.950
Unknown	1.32 (0.86–2.05)	0.204	0.90 (0.33–2.10)	0.829	0.16 (0.01–2.66)	0.201	1.11 (0.64–1.88)	0.709	1.06 (0.63–1.73)	0.829

*P-value is statistically significant

*BSI* Blood stream infection, *UTI* Urinary tract infection, *GII* Gastro - intestinal infection, *CBPN* Common bacterial pathogens of nasopharynx

Only age > 12 months was significantly associated with GII (*OR* = 2.9, 95% *CI*: 1.9–4.6, *P* < 0.001). Acorrelation between UTI or CBPN and the risk factors studied was not found.

## Discussion

This study demonstrated that *P. falciparum* causing malaria is still the main pathogen identified in febrile children in the Nanoro region in Burkina Faso. As malaria can be relatively easily diagnosed, under-or overtreatment of this disease could be avoided if health workers adhere to the results of diagnostic testing [6, 31]. Next to malaria, a very small proportion of the febrile malaria-positive children were also co-infected with another potentially fever causing pathogen, such as a bacterial bloodstream infection (bBSI), which can cause a prolonged fever after successful malaria treatment. More importantly, if malaria is ruled out as the attributable cause of fever, the alternative cause fever should be established taking into account that in about 20% of the febrile malaria-negative children the actual cause of fever could either be a bBSI or a viral gastro-intestinal infection (vGII) or a urinary tract infection (UTI).

The prevalence and distribution of possible causes of fever found in the present study are in line with other studies performed in Sub-Sahara Africa in the same age group [29, 32–37]. Importantly, the study re-emphasizes that a proper diagnosis of the cause of the fever episode in children in this age group followed by appropriate treatment is needed, as it will obviously save lives and prevent incorrect prescription of drugs. Only malaria can easily be diagnosed as a possible cause of fever in most health facilities in low and middle income countries at present. Therefore, there is a pressing need to develop practical tools (point-of-care or near point-of-care) to diagnose other treatable causes of fever in order to reduce morbidity and mortality in this age group [6].

Non-malaria infections that probably contributed to fever in this study were bBSI, UTI and vGII. The prevalence of bBSI was, despite differences in study designs, comparable to those reported in other studies in SSA (4 to 10%) [32, 38–40]. The severely ill cases were referred to the referral hospital Centre Medical Saint Camille. This explains why bBSI and UTI cases were more prevalent in the referral hospital than in the rural health facilities. The high prevalence of Gram negative bacteria in general, and non-typhoid *Salmonella* in particular, is in agreement with a previous study in the same area, and with the global trend [41]. UTI found in this study was less prevalent than reported in studies from Tanzania and Nigeria. However, the predominant bacteria causing UTI in Nigeria and Tanzania were *E. coli* (as in this study) [22, 28, 42–44]. In general, the hygienic conditions and sanitation are relatively good in the present study area and children are often well supervised by their parents or guardian, which could explain the low prevalence of UTI observed in this study. Around 5% of the febrile children were infected by vGII caused by rotavirus and adenovirus. This proportion is lower than that previously reported (33.8%) in Ouagadougou before the introduction of the rotavirus vaccine in Burkina Faso in 2013 [36]. The low prevalence observed in the current study could therefore be attributed to the rotavirus vaccination program in place in Burkina Faso. Contamination of stool cultures was not reported. However, 6.3% (32/507) of the urine samples collected by parents or guardians were contaminated. Urine collection is a rather difficult process, in particular to avoid contamination, and parents/guardians were not trained to do this and this explains the relative high rate of urine contaminations. Only 0.4% (3/684) of the blood samples collected by the nurses were contaminated.

This study also identified the presence of pathogens that can be considered as “non-probable cause of fever”, such as gastro-intestinal parasites, common bacterial pathogens of the nasopharynx and bacteria in stool. Still these pathogens require attention in patient management as they may affect health, for example in case the protective mucosal barrier of the intestine or nasopharynx is broken and they enter the blood stream [30, 45]. A limitation of the study is that viral respiratory tract infections, which are known to be a cause of fever [13, 21–23, 46–48] were not studied. These viral infections of the respiratory tract do not necessarily need treatment, and their detection require sophisticated laboratory facilities. As other studies have demonstrated that respiratory tract viral infections can be a major attributor to fever [21–23], it might be possible that these pathogens are the cause of febrile illness in the children in which no probable causes of fever could be identified. The study revealed some correlations between the presence of an infection investigated and the patient’s demographic, clinical and laboratory data. In particular a relation between having malaria and moderate to severe anaemia was found, which is in line with previous findings in Nigeria and Ghana [49, 50]. Furthermore, children with a normal WBC had a higher risk of being infected with malaria than those having a high WBC [51]. A high temperature or high WBC counts was not predictive for a bacterial infection in the present study, which is in contrast to previous findings [39]. Significant other correlations were not found.

## Conclusions

This study showed that malaria remains strongly associated with paediatric fever episodes in Nanoro. A relatively high number of non-malaria infections was found in malaria microscopy positive as well as malaria microscopy negative febrile children. Among these non-malaria infections, a “probable cause of fever” was mainly found in the malaria-negative febrile children. As treatment of several of these non-malaria infections is possible, there is a need for a practical tool to help clinician to screen for treatable causes of the febrile episodes. In the absence of such a diagnostic tool, malaria diagnostic test can be of help for the clinician to decide who should receive additional treatment, but over-or under-treatment will remain likely and puts febrile children at significant risk of morbidity and mortality.

## Additional file

Additional file 1: Multilingual abstract in the five official working languages of the United Nations. (PDF 320 kb)

## Abbreviations

bBSI: bacterial bloodstream infection
bGII: bacterial gastro-intestinal infection
CBPN: Common bacteria pathogen of nasopharynx
CFU: Colonies forming units
CRF: Case report form
CRUN: Clinical research unit of Nanoro
EDTA: Ethylene diamine tetra acetic acid
EMB: Eosin methylene blue
EPI: Extended program of immunization
Log: Logarithm
pGII: parasitic gastro-intestinal infection
*SD*: Standard deviation
*SS*: *Salmonella* and *Shigella*
SSA: Sub-Saharan Africa
STGG: Skim milk-tryptone-glucose-glycerol
TH: Todd-hewitt
UTI: Urinary tract infection
vGII: viral gastro-intestinal infection
WBC: White blood cells
WHO: World Health Organization
