# Supplementary material for: Treatable causes of fever among children under five years in a seasonal malaria transmission area in Burkina Faso
Source: Infect Dis Poverty. 2018 May 31;7:60. doi: 10.1186/s40249-018-0442-3 (PMC5994647; doi:10.1186/s40249-018-0442-3)
Supplement: Supplementary file 1 — Multilingual abstract in the five official working languages of the United Nations. (PDF 320 kb) [file 40249_2018_442_MOESM1_ESM.pdf]

Translation of the abstract into the five official working languages of the United Nations

أسباب الحمى القابلة للعلاج لدى الأطفال الأصغر من 5 سنوات في منطقة نقل للملاريا الفصلية، نانورو، بوركينا فاسو

فرانسوا كييمدي، ملرك كريستيان تاهيتا، بالبوغيني لومبو، توسانت روامبا، أثاناس م. سوم، هاليدو تنتو، بترا ف. منس، هنك، د. ف. هـ. شالنغ ومايكل بويل فان هنزبروك

موجز

الخلفية: لا تزال الحمى مشكلة صحية عامة رئيسية. في بوركينا فاسو، يعتبر أكثر من نصف الأطفال المصابين بالحمى غير مصابين بعدوى الملاريا. أجرت هذه الدراسة تقييما مستقبليا للأسباب المحتملة (القابلة للعلاج) للحمى لدى الأطفال في بوركينا فاسو.

الأساليب: أجريت دراسة استطلاعية بين الأطفال المصابين بالحمى (≤ 37.5 درجة مئوية) ممن تقلّ أعمارهم عن 5 سنوات في 4 مرافق صحية وأحد مستشفيات الإحالة الريفية في بوركينا فاسو. أخذت عينة من دم كل مشارك، بغرض الفحص المجهري عن الملاريا ولإجراء الزرع، وجمع البول لاجراء فحص الغميسة ولزراعته إذا كانت نتيجة اختبار الكريات البيض والنتريت إيجابية، وأخذت عينة من البراز لتحري وجود فيروس روتا / الفيروس الغدّي، وللزرع والفحص الطفيلي، كما أخذت مسحة من البلعوم لزرعها.

النتائج: أدرجت الدراسة ما مجموعه 684 طفلا مصابا بالحمى. عثر على ملاريا المتصوّرة المنجلية لدى 49.7٪ من المشاركين (684/340) وعلى حالات عدوى غير الملاريّة لدى 49.1٪ (684/336) من الأطفال. شملت حالات العدوى غير الملارية حالات العدوى المعدية المعوية (37.0٪)، وعدوى البلعوم الأنفي الناجم عن العوامل الممرضة البكتيرية الشائعة (24.3٪)، والعدوى البكتيرية في مجرى الدم (6.0٪)، وعدوى المجاري البولية (1.8٪). وكان نحو 45٪ (340/154) من الأطفال المصابين بالملاريا مصابين أيضا بعدوى غير ملاريّة، ولكن 3.2٪ فقط (340/11) من هذه الإصابات المشتركة أمكن اعتبارها سببا بديلا محتملا للحمى. في المقابل، لدى الأطفال الذين كان فحص الملاريا المجهري سلبيا لديهم، أمكن اعتبار 18.0٪ (344/62) من حالات العدوى هي السبب المحتمل للحمى. لم يتم عزل العوامل الممرضة من 23.7٪ (684/162) من الحالات المصابة بالحمى.

الاستنتاجات: لا تزال الملاريا هي أكثر الممرضات شيوعا بين الأطفال المصابين بالحمى في بوركينا فاسو. ومع ذلك، فقد كان عدد كبير نسبيا من الأطفال المصابين بالحمى مصاب أيضا بحالات عدوى غير ملارية. ويشكل وضع التشخيص الصحيح لهذه الحالات من الحمى غير الملارية مصدر قلق كبير، وثمة حاجة ملحة لتطوير المزيد من اختبارات نقطة الرعاية التشخيصية (POCT) ومن القدرات اللازمة لتحديد وعلاج أسباب هذه الحالات من الحمى.

Translated from English version into Arabic by Lina SM, through

TRANSLATORS
WITHOUT BORDERS

布基纳法索季节性疟疾传播区 5 岁以下患儿发热的可医治因素

Francois Kiemde, Marc Christian Tahita, Palpouguini Lompo, Toussaint Rouamba, Athanase M. Some, Halidou Tinto, Petra F. Mens, Henk D. F. H. Schallig and Michael Boele van Hensbroek

摘要

引言：发热仍然是一个重要的公共卫生问题。在布基纳法索，一半以上的发热患儿并非感染了疟疾。本研究前瞻性地评估了引起布基纳法索患儿发热的潜在（可医治）因素。

方法：在布基纳法索乡村的 4 家医疗机构和一家转诊医院，我们开展了对 5 岁以下发热患儿（≥37.5°C）的前瞻性研究。采集每位参与者的血液进行疟疾显微镜检查和培养；采集尿液进行试纸检测，如果检测发现白细胞和亚硝酸盐呈阳性，则进行培养；采集粪便进行轮状病毒/腺病毒检测、培养以及寄生虫学检测；采集鼻咽拭子用于培养。
结果：本研究共纳入 684 名发热患儿。49.7%（340/684）的患儿患有恶性疟，49.1%（336/684）的患儿不属于疟疾感染。非疟疾感染者包括胃肠道感染（37.0%），鼻咽部常见细菌性病原体（24.3%），细菌性血液感染（6.0%）以及尿路感染（1.8%）。近 45%（154/340）的疟疾患儿共患其他疾病，但只有 3.2%（11/340）的共同感染可被视为可能的替代性发热原因。相反，在疟疾显微镜检查中呈阴性的患儿中，有 18.0%（62/344）可认为是感染导致了发烧。23.7%（162/684）的发热病例没有分离到病原体。
结论：疟疾仍然是布基纳法索发热患儿中最常见的病原体。然而，相当多的发热患儿患有非疟疾感染。正确诊断非疟疾发热也是一个亟待解决的问题，迫切需要开发更多的及时诊断测试，提升确定和诊治非疟疾发热的能力。

Translated from English version into Chinese by Xue-Jiao Ma, edited by Jin Chen

CDC CHINESE CENTER FOR DISEASE CONTROL AND PREVENTION
NATIONAL INSTITUTE OF PARASITIC DISEASES

Causes traitables de fièvre chez les enfants de moins de 5 ans dans une zone de transmission saisonnière du paludisme : Nanoro au Burkina Faso

François Kiemde, Marc Christian Tahita, Palpouguini Lompo, Toussaint Rouamba, Athanase M. Some, Halidou Tinto, Petra F. Mens, Henk D. F. H. Schallig et Michael Boele van Hensbroek

Résumé

Contexte : La fièvre reste un problème majeur de santé publique. Au Burkina Faso, un enfant fébrile sur deux n’est pas considéré comme infecté par le paludisme. Notre étude évalue de manière prospective les causes (traitables) probables de fièvre chez les enfants burkinabés.
Méthodes : Une étude prospective a été menée auprès d’enfants (≥ 37,5 °C) âgés de moins de 5 ans présentés dans un état fébrile dans 4 dispensaires et un hôpital de référence d’une région rurale au Burkina Faso. Pour chaque participant, nous avons prélevé des échantillons de sang (recherche du parasite au microscope et culture), d’urine (tests par bandelette réactive et culture si positif pour leucocytes et nitrite) et de selles (recherche de rotavirus/adénovirus, culture et parasitologie), ainsi qu’un écouvillonnage nasopharyngé (culture).
Résultats : Au total, 684 enfants fébriles ont été inclus dans l’étude. Un paludisme à *Plasmodium falciparum* a été décelé chez 49,7 % (340 sur 684) des participants et des infections non palustres chez 49,1 % (336 sur 684). Les infections non palustres comprenaient des infections gastrointestinales (37,0 %), des rhinopharyngites bactériennes banales (24,3 %), des bactériémies (6,0 %) et des infections urinaires (1,8 %). Près de 45 % (154 sur 340) des enfants infectés par le paludisme présentaient une co-infection non palustre, mais qui ne pouvait être considérée aussi comme une cause de fièvre que dans 3,2 % des cas (11 sur 340). En revanche, chez les enfants négatifs pour le paludisme à l’examen microscopique, 18,0 % des infections (62 sur 344) pouvaient

être la cause probable de la fièvre. Aucun agent pathogène n'a été isolé chez 23,7 % enfants fébriles (162 sur 684).
Conclusions : Bien que le paludisme reste l'infection la plus fréquente chez les enfants fébriles au Burkina Faso, un nombre relativement élevé de ceux-ci présentaient des infections non palustres. Le diagnostic correct de ces fièvres non palustres est un problème majeur et il est urgent de développer d'autres tests de diagnostic sur le lieu des soins et les capacités d'identifier et de traiter leurs causes.

Translated from English version into French by Suzanne Assenat, through

Поддающиеся лечению причины лихорадки среди детей младше 5 лет в зоне распространения сезонной малярии Наноро в Буркина-Фасо.

Франсуа Кимде, Марк Кристиан Тахита, Палпугини Ломпо, Туссен Руамба, Атанас М. Сом, Халиду Тинто, Петра Ф. Менс, Хенк Д. Ф. Х. Шаллиг и Майкл Боле ван Хенсбрук

Аннотация

Краткое описание. Лихорадка по-прежнему остаётся серьёзной проблемой в сфере общественного здравоохранения. В Буркина-Фасо более половины детей с синдромом лихорадки не считаются заражёнными малярией. В этом перспективном исследовании оценивались вероятные (поддающиеся лечению) причины лихорадки у детей в Буркина-Фасо.
Методы. Среди детей младше 5 лет с синдромом лихорадки (≥ 37,5 °C) было проведено перспективное исследование в 4 медицинских учреждениях и одном лечебно-диагностическом центре в сельской местности Буркина-Фасо. У каждого участника были взяты образцы крови для микроскопии и посева на малярию, мочи для анализа тест-полосками и посева в случае положительного результата анализа на наличие лейкоцитов и нитритов, кала для анализа на ротавирус / аденовирус, посева и паразитологического анализа, а также мазок из носоглотки для посева.
Результаты. В исследовании приняло участие в общей сложности 684 детей с синдромом лихорадки. Малярийный паразит *Plasmodium falciparum* был обнаружен у 49,7% (340/684) пациентов, и инфекции, не связанные с малярией, у 49,1% (336/684) детей. Среди инфекций, не связанных с малярией, были обнаружены желудочно-кишечные инфекции (37,0%), общие бактериальные патогены носоглотки (24,3%), бактериальные инфекции кровотоков (6,0%) и инфекции мочеполовой системы (1,8%). Почти 45% (154/340) детей, инфицированных малярией, были коинфицированы инфекциями, не связанными с малярией, но только 3,2% (11/340) этих коинфекций можно было рассматривать в качестве возможной альтернативной причины лихорадки. При этом в микроскопии на малярию у детей с отрицательными

результатами в 18,0% случаев (62/344) вероятной причиной лихорадки могли быть инфекции. Возбудители заболеваний не были изолированы в 23,7% (162/684) случаев.
Выводы. Малярия остаётся наиболее распространённым патогеном, обнаруженным у детей с синдромом лихорадки в Буркина-Фасо. Однако относительно большое количество детей с синдромом лихорадки были заражены инфекциями, не связанными с малярией. Правильный диагноз этих не связанных с малярией заболеваний является серьёзной проблемой, и существует настоятельная необходимость в разработке большего количества диагностических анализов в местах оказания медицинской помощи и развитии мощностей для выявления и лечения причин лихорадки.

Translated from English version into Russian by Oksana Rozhko and Natalia Potashnik, through

TRANSLATORS
WITHOUT BORDERS

Las causas tratables de fiebre entre los niños menores de cinco años en una área de transmisión de malaria estacional, Nanoro en Burkina Faso.

Francois Kiemde, Marc Christian Tahita, Palpouguini Lompo, Toussaint Rouamba, Athanase M. Some, Halidou Tinto, Petra F. Mens, Henk D. F. H. Schallig y Michael Boele van Hensbroek

Resumen
Antecedentes: La fiebre sigue siendo un grave problema de salud pública. En Burkina Faso no se considera a más de la mitad de los niños con un cuadro febril como infectados por malaria. Este estudio prospectivo evalúa las causas probables (tratables) de la fiebre en los niños burkineses.
Métodos: Se llevó a cabo un estudio prospectivo entre niños menores de cinco años con cuadro febril (≥ 37,5 °C) que se encontraban en cuatro puestos sanitarios y en un hospital de referencia en la zona rural de Burkina Faso. Se tomaron muestras de sangre de cada participante para un diagnóstico microscópico de malaria, se hicieron exámenes de orina con tirillas reactivas y cultivo para obtener resultados positivos en leucocitos y nitrito, exámenes de heces para pruebas de rotavirus/adenovirus, cultivo y parasitología, y una recolecta de muestras nasofaríngeas para cultivo.
Resultados: En el estudio se incluyeron un total de 684 niños con fiebre. *El plasmodium falciparum*de la malaria se encontró en el 49,7 % (340 / 684) de los participantes y en infecciones que no eran malaria en el 49,1 % (336 / 684) de los niños. Entre las infecciones que no eran malaria estaban las siguientes: infecciones intestinales (37,0 %), patógenos bacteriales comunes en la nasofaringe (24,3 %), infecciones bacteriales del torrente sanguíneo (6,0 %) e infecciones del tracto urinario (1,8 %). Casi el 45 % (154 / 340) de los niños infectados con malaria también estaban infectados con infecciones no relacionadas con la malaria. Sin embargo, solo el 3,2 % (11 / 340) de estas otras infecciones podían considerarse como una causa alternativa posible de fiebre. Por el contrario, en los niños cuyo análisis microscópico había dado negativo en malaria, el 18,0 % (62 /

344) de las infecciones podían ser la causa probable de fiebre. Los patógenos no se aislaron en el 23,7 % (162 / 684) de los casos febriles.

Conclusiones: La malaria sigue siendo el patógeno más común encontrado en niños con fiebre en Burkina Faso. Sin embargo, un relativo alto número de niños con fiebre tenían infecciones no relacionadas con la malaria. El diagnóstico correcto de estas fiebres no relacionadas con la malaria es una gran preocupación, y existe una urgente necesidad de desarrollar máslugares de atención, pruebas de diagnóstico y medidas para identificar y tratar las causas de estas fiebres.

Translated from English version into Spanish by Cecilia Dragonetti and Estefanía Sánchez Rodríguez, through

TRANSLATORS WITHOUT BORDERS
